# College and Hospital Notes

**Published:** 1900-07

**Authors:** 


### COLLEGE AND HOSPITAL NOTES.

The Albany Medical College held its 69th annual commencement, Wednesday, May 2, 1900. Dr. Albert Vander Veer, dean, presided. Dr. James H. Canfield, librarian of Columbia University, delivered the address to the graduates. The class numbered 125, and was one of the largest and best ever sent out by this famous institution.

Dr. George Lenz, of Gloversville, was the class valedictorian, acquitting himself in a most creditable manner.

At the Alumni banquet Dr. Vander Veer acted as toastmaster. Dr. Thos. D. Crothers, '65, of Hartford, was elected president of the Alumni Association. Dr. Walter H. Conley, '91, of the Buffalo State Hospital, was in attendance at the meeting.

---

The Buffalo Fresh Air Mission Hospital, at Athol Springs, opened June 30, 1900. It is situated on the Lake Shore, ten miles from Buffalo, is reached in twenty-five minutes by train, and is most favorably placed for the treatment of infants and children suffering from cholera infantum and other diarrheal diseases. Physicians are urged to send to the hospital any case of cholera infantum or infantile diarrhea which would be benefited by country air. The attention of the profession is called to the fact that until the wards are filled with cholera infantum cases, children afflicted with any medical or surgical disease, not contagious, will be gladly received.

The hospital is free, is equipped with all necessary apparatus, and is provided with resident physicians and trained nurses. Information furnished by Dr. Irving M. Snow, 476 Franklin Street, or Dr. DeWitt H. Sherman, 666 Main Street, or at Fitch Hospital, 165 Swan Street. In urgent cases telephone the Fitch Institute.

---

The Batavia Hospital, of which mention was made in the Journal several months ago, is now an accomplished fact. A recent meeting of the Women's Hospital Association was held at which articles of incorporation were adopted as follows:

First—The name of the proposed corporation shall be the Woman's Hospital Association of Batavia, N.Y.

Second—The principal office of the proposed corporation shall be at Batavia.

Third—The number of directors of the proposed corporation shall be 15.

The fourth paragraph gives the names of the persons mentioned below, who are to serve as directors until their successors shall have been elected at the annual meeting of the proposed corporation.

Fifth—The annual meeting of the proposed corporation shall be held on the second Thursday in May at 3 o'clock in the afternoon on each and every year.

Sixth—All members of the proposed corporation shall be adherents of either the regular, homeopathic or eclectic systems of medical practice and the medical practice or treatment to be used or applied in such hospital shall be one or the other of the systems above mentioned.

There are already more than 100 members of the association and the following is the list of names of the incorporators: Mrs. T. J. Perfield, Mrs. S. A. Sherwin, Mrs. W. Harris Day, Mrs. Alice G. Fisher, Mrs. H. J. Burkhart, Mrs. M. Moynihan, Mrs. H. Young, Mrs. J. C. Baker, Miss Lydia M. Bates, Mrs. F. S. Wood, Mrs. J. S. Casey, Mrs. Hinman Holden, Mrs. C. A. Johnson, Mrs. Gardner Fuller and Mrs. A. E. Clark.

It is probable that a number of Buffalo physicians will be attached to the staff as consultants and specialists.

---

A DETENTION hospital is very much needed in Buffalo. This is an important port where hundreds of sailors are arriving and departing daily and when one becomes supected of small-pox or other contagious disease there is no place to assign him pending the development of the case, except to the pest house.

A good quarantine hospital with a detention department should be built without delay. Recently the Health Commissioner, Dr. Wende, has been obliged to quarantine two vessels on account of small-pox suspects, much to the loss of the ships, but there was no other way under the present conditions. Dr. Wende's views on the subject are pronounced and intelligent and should be listened to attentively by the Common Council.

